# Neonatal Intensive Care Unit to Home Discharge Communication: A Quality Improvement Project

**DOI:** 10.1097/pq9.0000000000000669

**Published:** 2023-07-10

**Authors:** Priyam Pattnaik, Suhas Nafday, Robert Angert

**Affiliations:** From the * Department of Pediatrics, Division of Neonatology, Connecticut Children’s Medical Center, Hartford, Conn.; † Department of Pediatrics, Division of Neonatology, The Children’s Hospital at Montefiore and Albert Einstein College of Medicine, Bronx, N.Y.; ‡ Department of Pediatrics, Division of Neonatology, New York University Langone Medical Center, New York, N.Y.

## Abstract

Neonatal intensive care unit (NICU) graduates require complex services after discharge. The NICU discharge process at Children’s Hospital at Montefiore-Weiler, Bronx, NY (CHAM-Weiler) lacked a system for routine primary care provider (PCP) notification. Here, we describe a quality improvement project to improve communication with PCPs to ensure communication of critical information and plans.

**Methods:**

We assembled a multidisciplinary team and collected baseline data on discharge communication frequency and quality. We used quality improvement tools to implement a higher-quality system. The outcome measure was the successful delivery of a standardized notification and discharge summary to a PCP. We collected qualitative data through multidisciplinary meetings and direct feedback. The balancing measures comprised additional time spent during the discharge process and relaying erroneous information. We used a run chart to track progress and drive change.

**Results:**

Baseline data indicated that 67% of PCPs did not receive notifications before discharge, and when PCPs did receive notifications, the discharge plans were unclear. PCP feedback led to proactive electronic communication and a standardized notification. The key driver diagram allowed the team to design interventions that led to sustainable change. After multiple Plan-Do-Study-Act cycles, delivery of electronic PCP notifications occurred more than 90% of the time. Surveys of pediatricians who received notifications indicated that the notifications were highly valued and aided in the transition of care for these at-risk patients.

**Conclusion:**

A multidisciplinary team, including community pediatricians, was key to improving the rate of PCP notification of NICU discharge to more than 90% and transmitting higher-quality information.

## INTRODUCTION

Approximately 77 of 1000 infants born in the United States are admitted to a neonatal intensive care unit (NICU).^1^ Newborns admitted to NICUs are at elevated risk of poor growth, chronic respiratory disease, and neurodevelopmental disabilities, particularly if they are extremely preterm and/or have very low birth weight.^2^ Many NICU graduates require chronic care management after discharge, including frequent primary care visits, multispecialty care, and community-based early intervention services.^3,4^ Studies in adults have indicated that a structured discharge plan tailored to each patient results in small reductions in hospital length of stay and readmission rates.^5^ However, in another study in adults, less than one-third of primary care physicians (12%–33%) could access their patients’ discharge summaries at the time of the first postdischarge visit.^6^ Poor communication can lead to negative outcomes, including discontinuity of care, compromised patient safety, inefficient use of valuable resources, patient dissatisfaction, and economic consequences.^7^ Outpatient physicians have estimated that their follow-up management is adversely affected in approximately 24% of cases because of delayed or incomplete discharge communications^8^ and have expressed dissatisfaction regarding these deficiencies.^9^

Before this project, no structured method of communication with primary care providers (PCPs) for NICU infants being discharged home was in place at the CHAM-Weiler NICU. Discharge plans for patients hospitalized for months and requiring multiple follow-up appointments, medications, laboratory studies, and/or medical equipment were not reliably communicated to PCPs. Consequently, the necessary details to care for NICU-discharged infants with complex cases could easily be lost. In our context, the extent of this problem was unknown, but individual reports of discontent led us to examine the problem and attempt to improve our outcomes. Because PCP notification (PCPN) should be universal practice, and approximately 2% of patients are transferred to rehabilitation centers where extensive communication is already established, we aim to achieve 90% PCPN.

This quality improvement (QI) project initiated communication with receiving physicians in the community to ensure relaying of critical information and care plans through the electronic medical record (EMR). In a prior study of computer-generated summaries, approximately 70% of physicians preferred this format, indicating that structured summaries were shorter and clearer than dictated ones.^10^ An EMR makes communicating medically pertinent data to PCPs easy and effective.

## SMART AIM

We aimed to improve PCP EMR notification of discharge from the CHAM-Weiler NICU from 0% to 90% between January 2017 and April 2018.

## METHODS

**Fig. 1.**
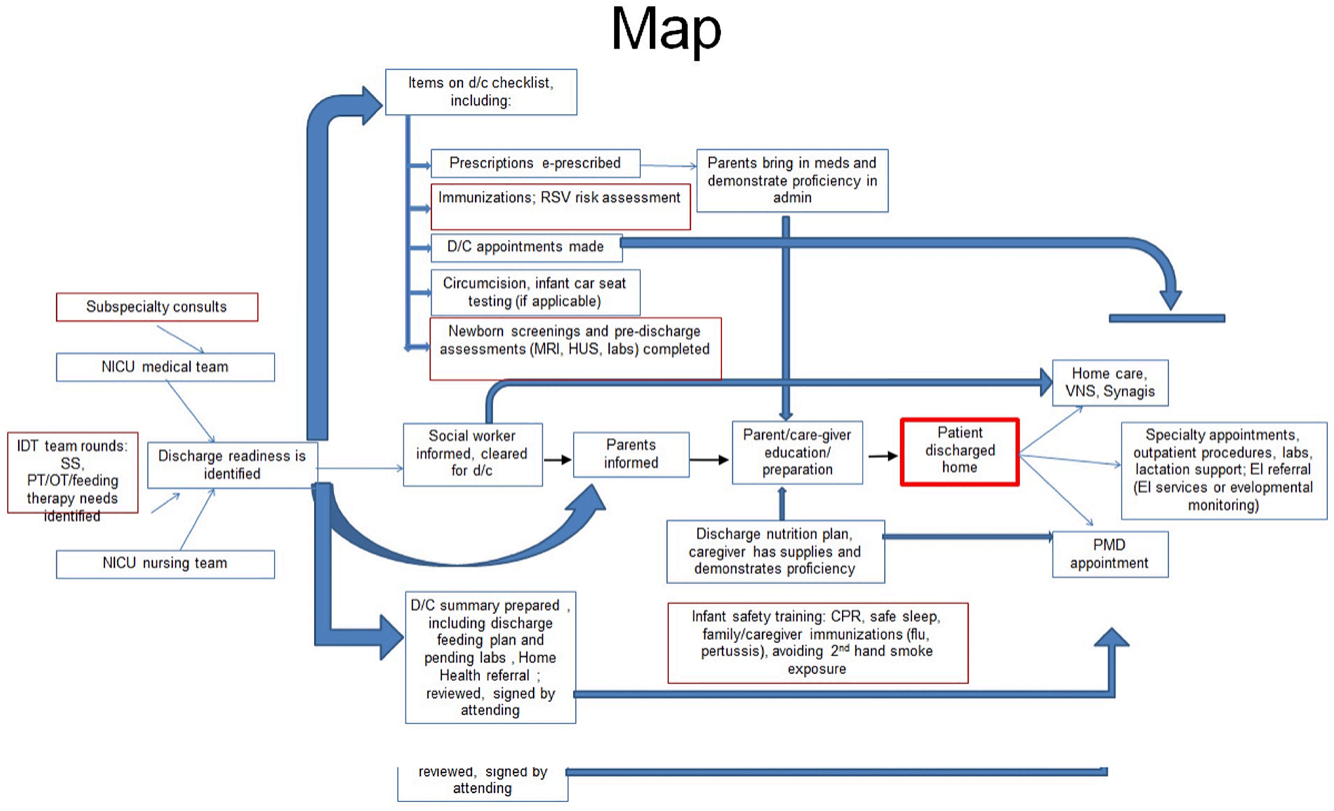
NICU discharge process map.

**Fig. 2.**
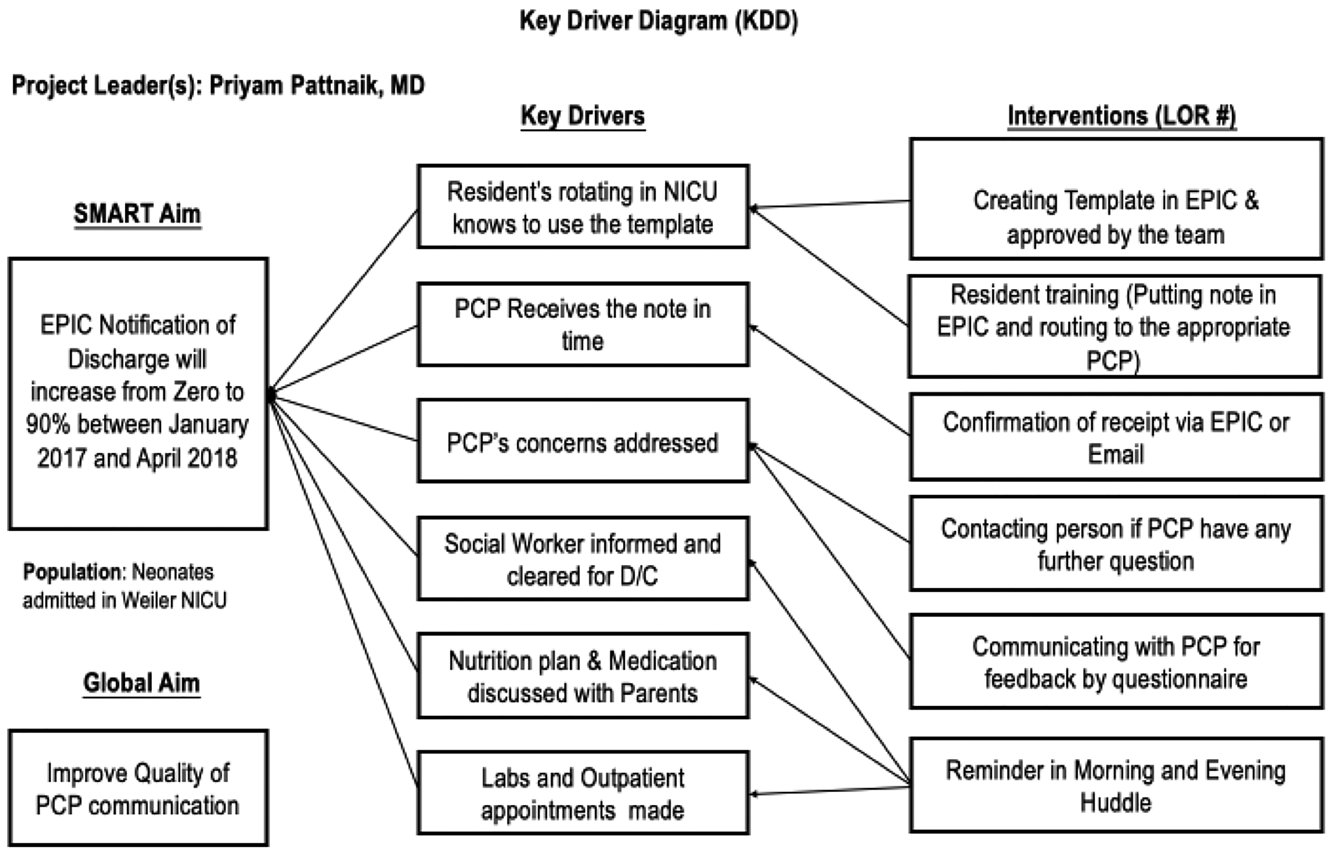
Pilot data key driver diagram.

CHAM-Weiler is a level IV NICU, quaternary care referral center located in an urban setting, with an average daily census of 37 and an annual admission rate of 700. We assembled a multidisciplinary team of key stakeholders and secured leadership support. The team included attending physicians, residents, advanced practice practitioners, nurses, primary care pediatricians, social workers, information technology specialists, and unit secretaries. We designed the project to align with the institutional goals of improving transitions in care. To achieve the desired workflow and culture changes, we used IHI QI tools, including creating a workflow diagram, a key-driver diagram, multiple Plan-Do-Study-Act (PDSA) cycles, and a run chart. We collected feedback through a PCP survey questionnaire to improve our discharge PCP communication. We implemented the tests of change on infants admitted to our NICU. In addition, we created a discharge map (Fig. 1) to understand the discharge process better and created a key driver diagram (Fig. 2), which was an integral part of this QI project.

## INTERVENTIONS

### Development of the PCPN Template

The QI group included PCPs from the primary care group who assumed care for NICU patients discharged home. The PCPs reported reviewing the discharge summary on the day of the infant’s outpatient visit or the night before. Most PCPs indicated that reviewing the lengthy discharge summary of prolonged NICU courses was time-consuming and exhaustive. Therefore, to achieve more efficient communication, they suggested that a short template containing all relevant medical information be sent to PCPs on the day of discharge or several days before discharge in very complicated cases and extremely low birth weight infants, given that newborns have outpatient appointments with their pediatricians 2–3 days after discharge. Their suggestions provided a basis for developing the PCPN template in the EMR, which the multidisciplinary team subsequently approved. We designed the template to automatically extract available data through a “smart phrase” created within the Epic EMR (Epic Systems, Verona, WI). The template contained the PCP’s name, date of discharge, date of PCP follow-up appointment, gestational age at birth in weeks, corrected gestational age in weeks at discharge, birth weight, and current weight. In addition, the template contained problems encountered in the NICU with active problems, scheduled follow-up appointments with subspecialties (if any), current medications, current nutrition, plans to follow-up on growth velocity and adjust calories, planned laboratory and imaging examinations, and the contact information of the NICU provider in the event of any further questions. The complete discharge summary was sent via the EMR as an attachment with this template.

A new set of interns and residents rotate each month in this academic NICU. In their orientation training, residents and interns were instructed to use the PCPN from the smart phrase and route the PCPN to the respective PCP at the beginning of their rotation. After the notification was sent, the note was delivered to the PCP’s inbox in the EMR. Confirmation of receipt of the PCPN was recorded within the system after the recipient read the communication. One patient was identified who was ready for discharge and scheduled to follow-up with a PCP within Montefiore Health Systems (MHS). A senior resident was trained to send the PCPN via EMR. After the PCPN was sent, the PCP was contacted via email and asked for feedback. Based on the response to the PCP survey questionnaire, we solicited feedback from the PCPs regarding the timeliness and content of our EMR template. We collected data from the EMR by examining the day the PCPN was sent with the discharge date and follow-up appointment with PCP and whether the physician reviewed the PCPN before the infant’s clinic visit.

We met with team members to evaluate the feasibility and adequacy of PCPN in daily practice, including barriers and promoters.

### Subsequent PDSAs

For all discharged infants, we implemented PCPN to PCPs within MHS. Gradually, all neonatal health care providers received training, including the attending physicians, neonatal nurse practitioners, physician assistants, neonatology fellows, and incoming residents and interns. During the morning and evening huddles in the NICU, NICU providers received reminders to send a PCPN. Patient care coordinators trained nurses to ask for PCPN when patients were nearing discharge. Residents included a PCPN reminder in their hand-off to-do list. Nurses incorporated a PCPN reminder in each patient’s bedside discharge checklist, and the unit clerk reminded the team of PCPN at discharge. We obtained data weekly and evaluated the number of discharges per week to MHS and the number of PCPNs sent and reviewed by the PCP.

### Balancing Measures

The team monitored the correctness of guidance provided to the PCPs by asking an attending neonatologist to check all discharge summaries for the accuracy of information/guidance provided to the PCPs, to avoid any inadvertent errors. A sample of PCPs filled out a survey to detect any perceived waste of time caused by the new PCPN.

This project was a QI initiative using medical records and data abstraction from EMRs to improve communication from the NICU to PCPs. The execution of this initiative did not involve interventions other than the PCPN and introduced minimal risk. We obtained IRB approval for this project.

## RESULTS

Because no formal discharge communication process was in place before the intervention period, the baseline rate was 0%.

Our PCP survey revealed 67% of PCPs did not receive the initial notifications before infant discharge. Consequently, they were uncertain about the various subspecialists involved in the infant’s care and whether any outpatient laboratory examinations or imaging was necessary. In addition, PCPs without prior knowledge of the NICU stay spent substantial time gathering patient information. Therefore, according to the PCPs, earlier contact and having a well-prepared NICU discharge summary were helpful.

We successfully implemented our first PDSA on 1 patient and received positive feedback from the PCP. For our second PDSA, we increased the number of PCPNs to 5 patients. After this test was successful, all incoming interns, residents, and other health care providers, including neonatal nurse practitioners, physician assistants, fellows, and attending physicians, were educated on the new process. An important prerequisite was that all patients had a chosen PCP before discharge to enable the PCPN to be sent promptly.

We analyzed data on all infants discharged to PCPs within MHS weekly.

**Fig. 3.**
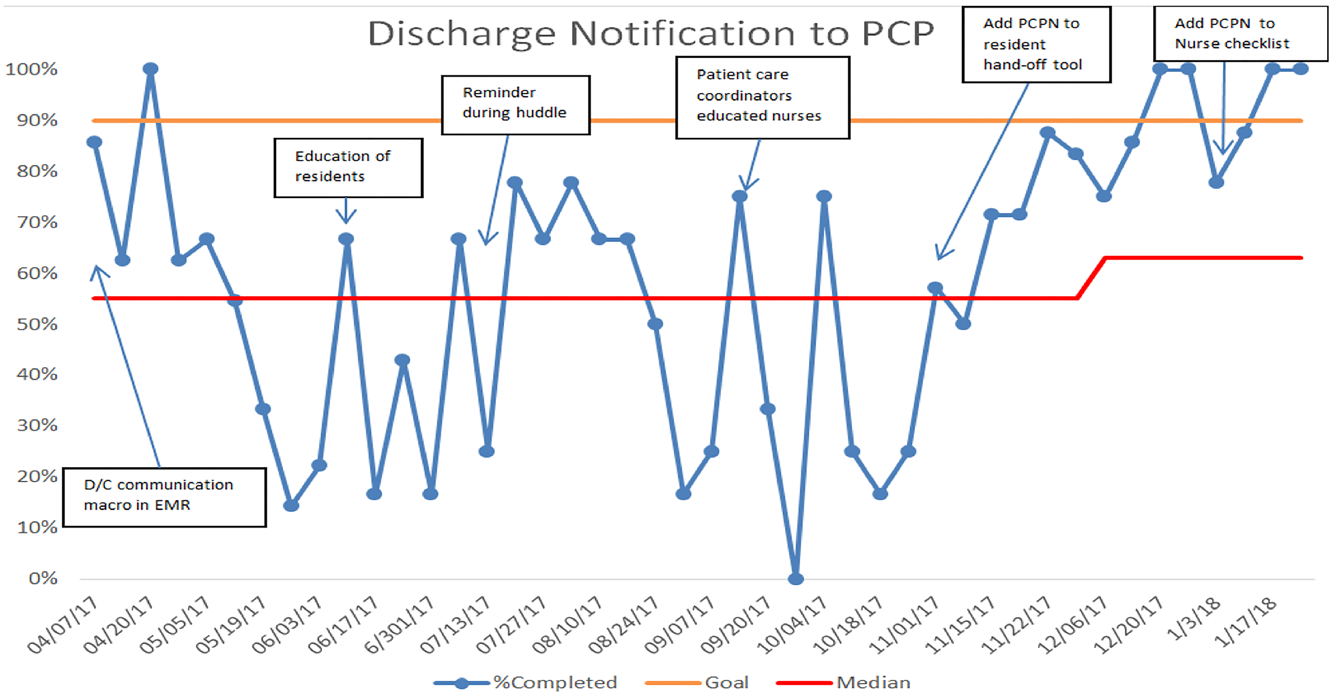
Pilot data run chart of PCPN within MHS.

We observed high variability in the PCPN rate, ranging from 0% to 100% in the first 6 months, with a median of 55% (Fig. 3).

We held another meeting with the team in the presence of nursing and physician leadership and senior residents, emphasizing the importance and timeliness of PCPN. Consequently, in subsequent PDSA cycles, we added PCPN reminders in hand-off to-do lists and the bedside discharge checklist and asked the unit clerk to remind staff to initiate PCPN at discharge. We analyzed a total of 669 discharges during the time frame of this project. Sixty-nine percent (n = 462) followed up with MHS physicians, whereas 31% (n = 207) followed up with non-MHS physicians.

Regarding the balancing measure, we observed no increase in the time required to prepare discharge, according to a qualitative survey of the personnel preparing the discharge summaries and completing the PCPN.

**Fig. 4.**
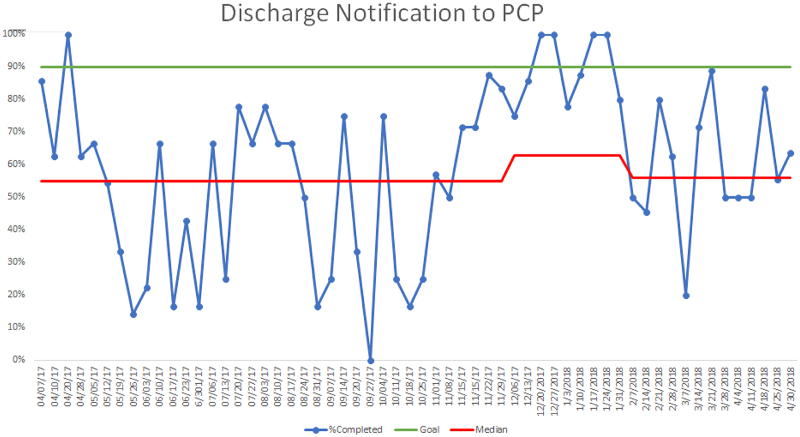
Pilot data run chart of PCPN to all PCPs including MHS and outside hospitals.

**Fig. 5.**
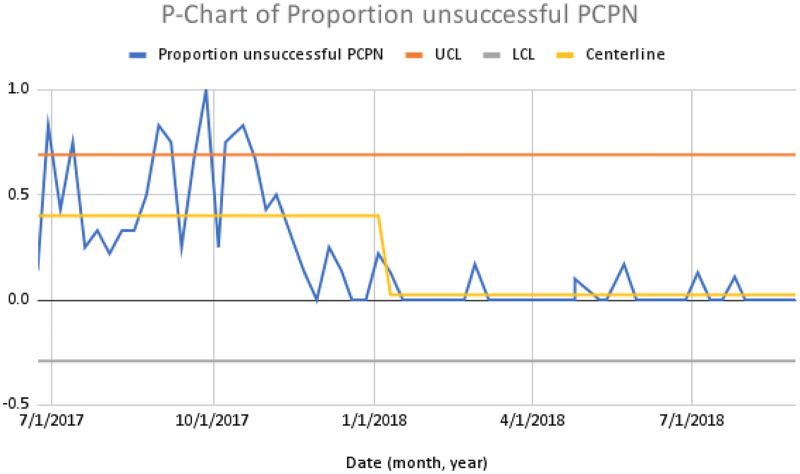
P-chart of the proportion unsuccessful PCPN.

We achieved a >90% rate of discharge PCPN to MHS pediatricians, with a median increase of 63% (Figs. 4, 5).

## DISCUSSION

Before this QI project, PCPs did not receive formal notifications after infants were discharged from the NICU, thus leading to inconsistent notifications and variable content. This project created a PCPN template in the EMR, which was implemented in January 2017. Through a series of PDSAs starting in March 2017, with data collected weekly, the PCPN rate increased significantly. The PCPN template contained critical elements of each patient’s history and plans for postdischarge care, including the contact information of the NICU provider in case of any further questions. Health care providers and residents rotating in the NICU were trained to use the template reliably. PDSA cycles facilitated adding the PCPN to the nurses’ discharge checklist to ensure compliance, sending reminder emails to residents, and requiring the unit clerk not to let infants leave without a copy of the template, which was routed to the PCP. With these interventions, we achieved a >90% rate of discharge PCPN to MHS pediatricians.

This QI intervention is, to our knowledge, the first describing use of the EMR to notify PCPs of NICU graduates. Several studies involving adults^11,12^ and children^13,14^ have emphasized the importance of communication with PCPs during the transition from the inpatient setting.^15^ The EMR is an easy and effective communication method with ambulatory care providers. Our interventions focused on using the EMR to convey the necessary medical information about infants’ NICU stays to their PCPs and routing the complete discharge summary. Sending only an exhaustive discharge summary of an infant’s complicated NICU stay was not time-efficient for PCPs. Moreover, poor communication during the transition of care could compromise patient safety if important information is missed. Using a template with the necessary data, which was sent along with the discharge summary, helped PCPs efficiently gather relevant information about infants’ NICU stays and outpatient recommendations.

Executing the PCPN template was challenging. We observed wide variability in the notifications in the first 6 months after introduction, and the highest rate occurred in months when the PI or coinvestigator was working in the NICU and could remind the team to send the template. We observed lower PCPN rates in months when the PI/coinvestigator was not in the NICU or when new residents started a rotation and were unaware of the process. Over multiple subsequent PDSAs, we addressed these barriers. Consequently, we improved our PCPN rate from 0% to >90% through better resident education, reminders, and meetings with senior nursing and physician leadership. However, the notification rate during the study period was below 100% because of several barriers. For example, some parents reported wanting to make the PCP appointments independently and to inform the NICU team immediately before discharge. In contrast, other parents took their infants to clinics covered by multiple providers, where a specific provider for the planned appointment could not be identified. In addition, discharge dates were difficult to predict for certain patient changes, such as monitoring for apnea of prematurity and weight gain concerns. Currently, the PCPN system performs excellently at MHS, with >95% compliance measured in our maintenance plan, thus highlighting that sustainability is critical for all QI interventions.

## LIMITATIONS

Our study has several limitations. First, we did not communicate with PCPs outside MHS during the study period. We addressed this limitation by using the EMR fax capability to notify non-MHS physicians; consequently, we achieved a >95% PCPN rate.

Because this QI initiative was a small-scale project limited to the NICU in a single hospital, its generalizability and feasibility require further study.

## CONCLUSION

Using the PCPN template in the EMR improved communication with PCPs at the discharge of neonates from the NICU by reliably and consistently providing the necessary data regarding the NICU stay and outpatient recommendations.

**SURVEY**

1-Do you get notified before the baby is discharged from Montefiore-Weiler NICU?

a. Yes
b. No

2-When do you get notified by the discharging team?

a. Not at all
b. Day of discharge
c. 1 week prior to discharge

3-How is the message communicated?

a. EPIC notification
b. Email
c. Telephone
d. Fax
e. Not Applicable

4-Are you informed about the NICU course of the baby?

a. Yes
b. No
c. Not Applicable

5-Are you informed about the various subspecialist involved in the baby’s care during his/her NICU stay?

a. Yes
b. No
c. Not Applicable

6-Are you informed about the follow up appointments?

a. Yes
b. No
c. Not Applicable

7-Do you get notified about the labs or imaging scheduled for the baby in the future?

a. Yes
b. No
c. Not Applicable

8-If you have any questions about the baby, do you get the contact information of the resident/fellow in NICU?

a. Yes
b. No
c. Not Applicable

9-Do you feel more confident in taking care of the baby if you have received prior notification?

a. Yes
b. No
c. Not Applicable

10-What information would you like to be communicated at the time of discharge? Please comment.

(If you don’t get notified please put not applicable)

## ACKNOWLEDGMENT

Assistance with the study: Neonatal attendings, fellows, pediatric residents, neonatal nurse practitioners, physician assistants, nurses, unit clerk Ms Fatima Macias of neonatal intensive care unit at CHAM-Weiler Hospital, working between January 2017 and April 2018.

## DISCLOSURE

The authors have no financial interest to declare in relation to the content of this article.
